# Marketing on a Shoestring Budget: A Guide for Small Museums and Historic Sites

**DOI:** 10.5195/jmla.2017.219

**Published:** 2017-04

**Authors:** Gerald Natal

Author Deborah Pitel is the marketing director for the Heritage Village Museum in Cincinnati, Ohio. She admits in the preface to her book that she had no experience in marketing when she was hired and so had to do some research to develop the necessary skills for the position. The books that she found on the subject of marketing for museums were written for larger institutions with in-house or hired marketing services in mind. She felt that the information contained therein was not realistically applicable to small museums like the Heritage Village.

The author’s trial-and-error efforts to improve attendance and visibility for her museum served as the impetus for writing this book, with the intention of helping others in similar situations. Even though *Marketing on a Shoestring Budget: A Guide for Small Museums and Historic Sites* was intended for small museums and historic sites, this book was reviewed for anyone who is working in any small or resource-challenged library and in particular from the vantage point of librarians in the areas of medicine and the health sciences.

*Marketing on a Shoestring Budget* is written in a conversational style and uses plain language in place of theoretical and college-level-course words such as “data mining,” “trend analysis,” “psychographics,” and “geodemography” that can be found in other books on marketing for special and academic libraries. This book does cover topics that are similar to those included in recent books to which it was compared, but the author strays from sales techniques and conventional marketing elements, which she says emphasize tangible products and the seller. She instead builds her marketing strategies around planning, public relations, and promotion, with a focus on the customer experience as an “intangible good.” This workable philosophy should be more easily relatable to libraries.

The first chapter begins with a good overview of marketing basics. Subsequent chapters offer practical advice on branding, target audience, advertising or media, websites, email, social media, blogging, and assessment of methods that those responsible for marketing in any type of library should find useful. Some information—such as concerns in the creation of marketing plans, suggestions for no-cost or low-cost software, and the importance of partnerships—will have more direct application than information on direct mailing or gender of website users, for instance. Each chapter ends with notes used for that chapter, along with a comprehensive bibliography. Many of the examples and resources cited are from blogs or other online sources that should be easily accessible to readers.

A visit to the website of the Heritage Village Museum provided evidence that the author practiced what she preached, as many of the principles and techniques discussed in her book were noticeable on the website. Even though other books on marketing for libraries exist, *Marketing on a Shoestring Budget* will deliver to readers the basics of marketing with practical suggestions for expanding patronage and improving the presence of libraries in an easy-to-understand format. If nothing else, the author’s journey should serve to inspire others with no marketing experience to improve awareness of their hospitals and medical or health sciences libraries.

